# Psychotic disorders of space and time – A contribution of Erwin W. Straus

**DOI:** 10.3389/fpsyt.2023.1150005

**Published:** 2023-04-11

**Authors:** Marcin Moskalewicz, Thomas Fuchs

**Affiliations:** ^1^Phenomenological Psychopathology and Psychotherapy, Psychiatric Clinic, University of Heidelberg, Heidelberg, Germany; ^2^Philosophy of Mental Health Unit, Department of Social Sciences and the Humanities, Poznan University of Medical Sciences, Poznań, Poland; ^3^Department of Ontology and Epistemology, Institute of Philosophy, Marie Curie-Sklodowska University, Lublin, Poland

**Keywords:** lived time, lived experience, depression, psychosis, phenomenology, temporal delusion, physiognomy, psychopathology

## Abstract

This paper presents and discusses a manuscript by one of the core founders of phenomenological psychopathology, Erwin W. Straus, concerning psychotic disorders of space and time (see attached Supplementary material). Written in June 1946, the manuscript is published for the first time as supplementary material to this paper. It is a clinical case-study of a patient with psychotic depression from Henry Phipps Clinic. It contains themes known from both Straus’ earlier and later work on lived time and mental illness, in particular a critique of physicalism in psychology, a vindication of primary sensing, a description of the spatiotemporal unity of lived experience, and the notion of temporal becoming. However, it is the only work by Straus that explores in such detail a patient’s case and shows how the lived experience is spatiotemporally structured and intrinsically related to affectivity, embodiment, and action. The manuscript is yet another piece of evidence of Straus’ significance in developing the field of phenomenological psychiatry in both Germany and the United States.

## Introduction: the manuscript and its author

This paper presents and discusses an unpublished manuscript by Erwin W. Straus titled “Psychotic Disorders of Space and Time” from 1946 (see attached Supplementary material) (1). Completed on a Sunday afternoon in June 1946, but certainly conceived earlier, the 58 typewritten pages long set of reflections oscillates around a clinical study of a patient with psychotic depression from Henry Phipps Clinic, where Straus was researching compulsive behaviors in the 1940s. It is a detailed exploration of a troubled subjectivity that shows a direct application and relevance of phenomenological categories for understanding and possibly treatment of depression. The descriptions of the patient’s unusual lived experience are woven into Straus’ more philosophical reflections concerning mostly the nature of normally lived space and time and his critique of physicalism in psychology in particular.

Erwin W. Straus belonged to the group of core founders of phenomenological psychopathology. A native of Germany, Straus studied medicine in Berlin and Munich, and begun his scientific carrier by defending a medical doctorate in 1919. He specialized in psychiatry under Karl Bonhoeffer and neurology under Richard Cassirer (2–4). Alongside luminaries, including Ludwig Binswanger, Viktor Emil von Gebsattel, and Jürg Zutt, he established the first and leading phenomenological psychiatry journal in Europe –Der Nervenarzt.

Straus has been concerned with the question of time since the very beginning of his career. In an early 1926 paper *On Individuality*, in the context of understanding biological and psychological theories of human development, he observed that: “Everything depends upon how time is conceived. We need a theory of time, which makes it possible to transform the mere succession of a temporal series into a relationship immanent to the things” (5). In another early article on depression from 1928, Straus suggested that distortions of the temporal structure of experience may be able to provide ground for uniting otherwise separated categories of mental disorders (6).

In 1938 Straus began his forced emigration to the United States, initially as a professor of psychology at Black-Mountain College in North Carolina (1938–1944), and then as a research fellow at John Hopkins University (1944–1946), where he worked on the *Psychotic Disorders of Space and Time* manuscript. Why the manuscript was never published remains unclear. One explanation might be that Straus never published patients’ case studies –and both *Psychotic Disorders of Space and Time* from 1946 and *Temporal Horizons* from 1952 (published in 2018) (7) are thematized around clinical cases. Another reason could be that both unpublished manuscripts contain a lot of philosophical ruminations that Straus considered unfinished. In both, Straus develops his original conceptual framework to address the lived experience of space and time by disputing the views of important intellectual figures, Hume and James in the 1946 paper, and Bergson and Freud in the 1952 paper. Also, many of the threads from the 1946 manuscript can be found in other works by Straus: the theme of future orientation of experience was already explored earlier (6), the thoughts on physiognomy appeared in Straus’ book *On Obsession* (8), some theoretical thoughts on time in his later *Chronognosy and Chronopathy* paper (9), and the patient’s depressive psychotic experiences were quite probably used in *Disorders of Personal Time in Depressive States* published a year later. Also, in 1946, Straus got a new job as a Director of Research and Education at the Veterans Administration Hospital in Lexington, Kentucky (1946–1961) as well as a Lecturer at the University of Kentucky (1946–1956). In Lexington, Straus kept working on the issue of lived time, on which he published three more papers (9–11). Neither of those, however, nor any of his unpublished works on this subject compares with the scope and comprehensiveness of the *Psychotic Disorders of Space and Time*. Straus traveled back to Germany several times but remained in Lexington, where he died in 1975.

## Spatio-temporal unity and disunity in psychopathology

At first sight, Straus’ patient, whose experiences are discussed in the manuscript, has a practical problem with lived space – he easily gets lost in a city that he nevertheless knows well. Simultaneously, he has some odd perceptual and illusion-like experiences regarding space. For example, some things appear to him as flat and remote, and others as big. A ball thrown seems to move in slow motion, and the ward clock is seen as no longer round. The patients’ symptoms are obvious to notice, but their underlying structure is hidden, as it were. These perceptual spatial problems, Straus argues, stem from more elementary and preconscious disturbance of space–time. The most explicit manifestation of this disturbance regarding time is the patient’s statement that time ceased to exist. But what does that actually mean?

Straus begins his reflections by explicitly departing from the intellectual tradition of psychology, which, in his view, is tainted with some hereditary deficiencies. The major one is the reduction of the lived experience of space and time to perception –a recurrent theme of Straus’ work despite his neuropsychiatric background. Perception of time has been traditionally addressed by chronometrical tasks, i.e., by exclusively conceiving it as abstract and ignoring more complex and hidden aspects of lived experience. Time cannot be an object of perception, Straus claims, for every perception has a temporal content. This temporal content is also not simply experiential but personal –it is related to one’s life history and values and meanings. For example, Straus asserts that it is the meaning of impressions in the context of personal becoming that makes time pass slowly or fast in perception. Emotions are also temporal to the extent that temporality and affects are two sides of the same coin. Characteristically, Straus frames this issue in biological terms of the spectrum of lived future possibilities extending between survival and annihilation, and their corresponding emotional states.

Already in his early book *Geschehnis und Erlebnis* (Event and Experience) from 1930, Straus asserted that lived time is a central problem of theoretical psychology (12). He criticized all psychological theories (including psychoanalysis) that assume a notion of time presupposed by psychophysiology and psychosomatics, which leads one astray –away from the phenomena and toward a limiting physicalist notion. Overall, in his early theory of lived time, Straus presented a view of the human being as capable of escaping the natural causality and tragic fatalism of the past (13). In the 1946 manuscript, Straus goes back to what he sees as the philosophical underpinnings of the “original sin” of psychology in the work of Hume and James, whom he reproaches for their alleged cinematic view of temporal experience. Regarding James, this comes as a surprise given that James famously wrote about the specious present. Straus argues, however, that James’ silent theoretical presupposition was psychological atomism that entailed a discontinuity of mental life. James allegedly could not free himself from the power of spatial motions and turned back to Hume’s view of time as based upon a sequence of impressions. Straus, like Husserl before him, uses the example of a melody to show that impression of a sequence is prior to the sequence of impressions. The same concerns space, as spatial experience is not constituted on the basis of summing some perceptual parts together. Any such addition presupposes a spatial whole as a frame of reference. On the other hand, drawing a representation of space, such as a map, is distinct from its lived experience. It requires dislocating oneself from the center and looking at the world from a detached perspective.

The curious thing about the patient’s delusion concerning the existence of time and his perceptual disturbance of space is that he claims that time has no beginning and no end for him, and that space is heavily deformed while actually *knowing* world time, *being aware* of the order of day and night, and *being capable* of symbolically orienting himself on a map. The patient thus loses the spatiotemporal perspective of lived experience (sensing), but not its abstract (numerical or geometric) representation (knowing). Unlike in aphasia, mentioned by Straus at the beginning, it is concrete and lived, and not abstract and schematic space that is primarily affected. Such split between sensing and knowing is a crucial psychopathological characteristics of the patient’s lived experience. Regarding time, it has been compared to schizophrenic double book-keeping (14). Straus’ patient presents such a split regarding both time and space, but it is temporality that appears as an underlying source of the disorder.

Straus saw these two aspects of lived temporality –which he called the immanent (or personal) time and the world (or clock) time –as originally intrinsically connected and not in ontological conflict (for more details on Straus’ unity view of time and different stages of his thinking on this subject see (15)).

In his later manuscript *Temporal horizons* (1952), Straus exemplified his unity view with the concept of “today” as pertaining to both orders of time, binding them together, as it were (7). In a reflective attitude toward time, there is always some degree of discordance and asynchrony between these two aspects, but in pathological situations this discordance intensifies. Such discordance, Straus claimed, is based on the cessation of advancing toward the future, which concerns the whole of personal becoming, and not just the conative dynamics. In the extreme state of depressive psychosis, the lived time comes to a standstill. It is experienced as unreal, even though one can still perceive the movement of the hands of the clock and count the passing days on the calendar. The two orders or time are now wholly incongruent.

Straus will repeat his ideas on the discordance between personal and objective time from the 1946 manuscript in a condensed version in his *Disorders of personal time in depressive states* published shortly after (16). The same core conception will pertain to Straus’ ideas on compulsive behaviors, though he will articulate the temporal discordance slightly differently. In his 1948 book *On Obsession* –the first published after moving to the United States – Straus explores obsession as a disturbance of sympathetic relation to the world (8). From a temporal angle, compulsivity consists of perfectionist expectations. While in daily life one typically leaves some room for the unknown, an obsessive person expects full certainty and thus has trouble dealing with the unpredictable. Trying to bring everything under control, one loses the continuity of life and experiences a sequence of present moments instead, as if living through the cinematic conception of temporal consciousness.

## Critical considerations on the I-world relationship

In the *Psychotic Disorders of Space and Time*, Straus frames the theme issue in terms of the changes in the primary structure of the I-world relations that are affected not only temporarily, but also spatially – a continuous theme he will also explore in his later piece *Norm and Pathology of I-World relations* (17). In the unpublished manuscript, Straus re-defines Koffka’s notion of physiognomic characters, which he grounds in the spatio-temporal structure of the “I-world relationship.” Straus’ departure point is phenomenological, but it is not focused solely on consciousness or being-there as intrinsically connected with the world. Unlike Martin Heidegger’s concept of Dasein’s being-in-the-world (18), Straus’ notion is about the most concrete, embodied, and animal existence (17). A trained neurologist, Straus, different from Heidegger, emphasizes the continuity between self and nature. Still, like Heidegger, he performs a devastating, life-world-based phenomenological critique of the Cartesian, objective psychology as falsely following the principles of a mechanistic (linear and causal-effective) account of time. Such psychology, Straus argues, considers consciousness as a thing among things in the world, moving in a body through geometrical space and clock time, and neglects its most concrete and lived, even if directly non-observable content.

In his 1946 comments on the physiognomic characters, Straus vehemently opposes any purely physicalist analogies that would lead to losing sight of the phenomena themselves. He argues that sensory experience and power relations in the environment are indivisible – an example of his patient’s deformed sense of the size of his physician. Physiognomic characters resemble in some aspects James Gibson’s later affordances (coined in the 1960s), but they are more basic and have an affective, atmospheric, not only sensorimotor character (e.g., the “obtrusiveness of smells” which Straus mentions on p. 16 as a “physiognomic phenomenon”). Straus sees physiognomies as embedded in the primary structure of I-world relationship, in which the potentiality of movement and power are always involved.

In the manuscript, Straus also mentions *conation* or organic drive – the fact that experiencing beings are ahead of themselves and see the world in terms of capabilities, attraction, and repulsion, so that perception and movement are two sides of the same coin, as contemporary neurophenomenology also underscores (19). Sensory experience thus appears intrinsically connected with mobility, which is why the patient also finds it difficult to dance. One prereflectively responds to physiognomies as appealing or appalling, and the loss of conation means a loss of potentialities for action. Mental illness thus deforms the patient’s lived space, inhibiting his responsivity and exchange with the environment (20).

These themes touched upon in the manuscript emerge from his earlier major work *Vom Sinn der Sinne* (published in English as *The Primary World of the Senses*), which presented the summary of his views on the anthropological foundations of psychology (21). The book was concerned with man’s pre-reflective attunement with the world and vindicating sensory experience in its pre-cognitive right. Accordingly, Straus’ concept of physiognomy points to the basal features of embodied experience and precedes much later discussion of situated enactive cognition, e.g., any experience is lived as attractive or threatening, supportive or resisting, to mention some of these features. The world is not faceless, and time and space are not abstract but lived and implicitly connected.

On this background, the basic alteration of physiognomic experience in psychosis can even undermine the sensorimotor affordance structure of the environment. As Straus points out:

*“With the physiognomic dismemberment of space, performance and perception of motion change accordingly. In a baseball game A. Br. was not able to aim correctly at a goal When he looked at a ball in light, he had an impression of discontinuity comparable to that of, slow motion’” (p. 17)*.

The reason for this lies in the fact that the structure of the experienced space is primarily not geometric-euclidean in nature, but dependent on the physiognomies of the environment as well as on the temporality of the experience: “Space, visible space, unfolds itself and is open to the future. Space is not a timeless order of places side by side; it is a field of potential action” (p. 17). Hence, if conation as the basis of the flow of subjective time comes to a standstill, then not only the future as a sphere of potential action closes off. Space also distances itself, and things move to an unreachable distance:

*“The ‘There’ is experienced in disconnected remoteness. The patients cannot reach beyond themselves; they cannot anticipate a goal” (p. 17)*.

Similar observations on the spatiality of endogenous-depressed patients have also been published 10 years later by Tellenbach (22). The perception of spatial depth as accessibility is lost; space becomes featureless, flat, and distant. The psychotic disorders Straus examines by means of his case study thus prove the *primary unity of conation, temporality, and spatiality* in human experience.

## An existential disorder?

Straus’ identification of the patient’s hidden spatio-temporal disturbance is based on his broader view of vital temporal becoming. The notion of disordered temporal becoming provides Straus with a diagnostic criterion that enables distinguishing endogenic depression from more psychogenic disorders. The criterion is whether the lived time is growing or declining, or immobile. From a critical angle, temporal growth and decline metaphors are spatial and binary, and they lack specificity. Straus speaks mostly of endogenic depression, but he also mentions the same syndrome of cessation of temporal becoming (which he calls, following Minkowski, *trouble générateur*) as pertaining to sexual neuroses and addictions. For example, Straus’ depressive patient lives in a continuous present, in which time flows amorphously, not unlike in addiction (23, 24).

The distinction Straus draws is between those mental disturbances, which are more neurotic, and in which future potentialities are preserved (even though their actualization is suspended), and those, in which the very potentiality is afflicted (as in the case of the psychotic depressive patient). In the latter case, a vital inhibition is claimed to stem from the very core of organic becoming. Straus tacitly argues here against psychodynamic theories of depression and in favor of his biologically grounded phenomenology. Hence, he underlines that some alterations of temporal becoming may be of biological origin but still affect interpersonal relations and conflict situations. The process of temporal becoming is vital, implying that lived time is biologically grounded. However, as Straus will explain in his last published paper on time from 1967, it is also always mediated by personal experience (11).

## Conclusion

The disorders of space and time are no longer rarely discussed in psychiatric literature, as Straus noticed in 1946. Almost 80 years later, they are a subject of considerable importance for phenomenological psychopathology (25). On the occasion of Straus’ 70^th^ birthday, Jürg Zutt wrote that the significance of Straus’ distinctive thinking about central problems of clinical psychiatry would be appreciated only in the future. It is a view with which we –commenting on his unpublished work from over seven decades ago –utterly agree (26).

There are many parallels between Straus’ and Minkowski’s, as well as Binswanger’s and von Gebsattel’s reflections on temporal experience in mental disorders, even if expressed in different terminologies. These similarities have already been attributed to the subject’s nature and commented upon (27). One of the key distinct aspects of Straus’ thinking is that he overcomes Jaspers’ restriction of phenomenology to pure descriptions and moves toward exploring the fundamental structures of lived existence. At the same time, unlike Ludwig Binswanger and Medard Boss, he does not succumb to Heidegger’s fundamental ontology and, unlike Minkowski, to Bergson’s metaphysics. Straus thinks for himself, and his analyses are always ‘sober’ and conceptually clear. At his time, he was perhaps a phenomenological psychopathologist to think in the most ‘medical’ way.

The unpublished 1946 manuscript is an evidence of this. What makes it unique is that, to the best of our knowledge, it is the only work by Straus that explores a patient’s case in such detail, and on its basis shows how the lived experience is spatiotemporally structured and intrinsically related to affectivity, embodiment, and action. In today’s 5E idiom, one would say that experience is ‘emotionally enacted’. Straus’ understanding of psychotic depression as an alternation of temporality and embodiment and their interpersonal embeddedness is thus very modern in its core, although not fully developed (28). The emphasis on physiognomy as underlying primary spatial experience and the variability of appearances though activity and movement is also original. Overall, Straus’ significance for developing the field of phenomenological psychiatry in both Germany and the United States is indisputable.

## Data availability statement

The original contributions presented in the study are included in the article/Supplementary material, further inquiries can be directed to the corresponding author.

## Author contributions

MM transcribed and edited the Erwin W. Straus’ manuscript and wrote the first draft of the article. TF corrected and amended the article. All authors contributed to the article and approved the submitted version.

## Funding

For the publication fee, we acknowledge financial support by Deutsche Forschungsgemeinschaft within the funding programme “Open Access Publikationskosten” as well as by Heidelberg University and Alexander von Humboldt Foundation. Research funded by National Science Center, Poland [No. 2021/42/E/HS1/00106] and supported by Alexander von Humboldt Foundation.


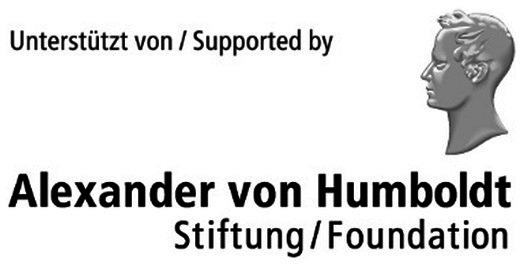


## Conflict of interest

The authors declare that the research was conducted in the absence of any commercial or financial relationships that could be construed as a potential conflict of interest.

## Publisher’s note

All claims expressed in this article are solely those of the authors and do not necessarily represent those of their affiliated organizations, or those of the publisher, the editors and the reviewers. Any product that may be evaluated in this article, or claim that may be made by its manufacturer, is not guaranteed or endorsed by the publisher.

## Supplementary material

SUPPLEMENTARY DATASHEET 1 Erwin. W. Straus, Psychothic disorders of space and time.
