# Supplementary material for: Psychotic disorders of space and time – A contribution of Erwin W. Straus
Source: Front Psychiatry. 2023 Apr 11;14:1150005. doi: 10.3389/fpsyt.2023.1150005 (PMC10126461; doi:10.3389/fpsyt.2023.1150005)
Supplement: SUPPLEMENTARY DATASHEET 1 — Erwin. W. Straus, Psychothic disorders of space and time. [file Data_Sheet_1.pdf]

Published as a supplementary material to:
M. Moskalewicz, T. Fuchs, Psychotic disorders of space and time – A contribution of Erwin W. Straus
Frontiers in Psychiatry 2023, https://doi.org/10.3389/fpsyt.2023.1150005

Erwin W. Straus

Psychotic disorders of space and time

edited by M. Moskalewicz*

A Neglected Subject

Space and time are basic forms of experiencing. Our experiences not only occur in time, they have – without exception – an inherent temporal content, and, if not all, certainly our sensory experiences are related to spatial orders. It is therefore to be expected that disorders of space and time would present themselves frequently in psychopathology. However, except for apraxia and agnosia, this topic is rarely discussed in psychiatric literature. If it is mentioned at all, usually one single aspect is taken into consideration: orientation and disorientation in space or time. In a survey of the "Experience of Time in Mental Disorder," Aubrey Lewis said: "one can still comfortably read through all the available literature, written by a handful of German and French psychiatrists, on time-phenomena in mental disorders." The situation has not changed much since 1932, the year in which Lewis presented his paper (Lewis, 1932).

Paul Schilder in one of his posthumous books dedicated one chapter to "Space" and another one to the "Psychology and Psychopathology of Time." Fifty pages supplied Schilder with enough room – in spite of ample quotations – at least to touch upon the main problems discussed in recent literature (Schilder 1942). Less rhapsodic reviews are to be found in two papers, read, in 1932, to the Swiss Psychiatric Society by Eugene Minkowski (1932-33) and Ludwig Binswanger (1933). In books by Minkowski (1933) and Binswanger (1942) the psychopathological phenomena are discussed on a broad basis, in context with the general philosophical and psychological

* The manuscript was transcribed and edited on the basis of its last version from 1946 preserved at Erwin. W. Straus Archive, Simon Silverman Phenomenology Center, Duquesne University, USA, Ref. FF 143 (© Simon Silverman Phenomenology Center, license CC BY-NC-ND 4.0). Since a number of corrections in this version was minimal, it was likely considered finished by Straus. The only major editorial change was adding the headlines, for which the editor takes full responsibility. In addition, obvious spelling and punctuation errors were corrected, footnotes were rearranged into the text to make the reading smoother, and references were remade into the APA style.

problems. The interest of the handful of German and French psychiatrists was aroused mainly by two of my own papers (Straus, 1928, 1930).

This taciturnity might have a very simple reason: that not much is said, because not much can be said; that disturbances of this kind are rare; that as basic forms time and space may be most resistant to a psychotic onslaught; and that, therefore, normal as well as pathological experiences usually develop within one and the same spatio-temporal framework. This reasoning and assumption to which it leads, expedient as it may be, are not well supported by the facts. Even if we did not know from experiments with drugs, like marijuana or mescalin, what deep alterations space and time may undergo, and even if we had never heard a schizophrenic patient describe the frightening change of the physiognomy of the world, remembrance of our dreams alone would hinder us from accepting this hypothesis. We have to look for better explanations.

In some cases which we all have seen, the disturbances of time and space were obvious enough. Yet only scanty reports were to be found in the case histories. The observers, not knowing what to make of these symptoms, neglected them in their descriptions. They treated them as merely casual accessories, not worth mentioning.

Disorders of space or time are not always so conspicuous that they cannot escape attention. As all our observations depend on our questions, we have to learn to question. The history of clinical symptomatology is by no means a history of discoveries of hidden phenomena. Certainly, many physicians had seen before Babinski the reflex which is now connected with this name. They had seen it, but they had not observed it. They took it as one of the many accidental variations of the positions of our limbs. The dorsal flexion of the big toe first became a symptom when Babinski observed its regularity, noted its clinical conditions and understood its pathophysiological meaning. In the behavior of our patients the typical is always mixed with the accidental. A machine which would faithfully record "every" utterance and gesture of a patient, a pan-recorder, as suggested by Lawrence Kubie, would be of no value. In observation and description we will always have to discriminate the meaningful from the insignificant. Our selection is determined in the last analysis by our understanding of

Published as a supplementary material to:
M. Moskalewicz, T. Fuchs, Psychotic disorders of space and time – A contribution of Erwin W. Straus
Frontiers in Psychiatry 2023, https://doi.org/10.3389/fpsyt.2023.1150005

meanings. It is the meaning which makes symptoms visible. If we knew more about the meaning of disorders of space and time, we should look out for them and see them more readily. Even so, our progress would still be delayed by two obstacles.

Geometric Space and Clock Time vs Experience of Space and Time

First when we speak about disturbances in the experience of space and time these terms make us think of the space of geometry and the time indicated by a clock. However, both these forms are abstract schemes. The experience of space and time is much more concrete. Space – I am now speaking about space only in relation to seeing – is experienced as a room, a street, a landscape, a finite space, covered by the sphere of the sky, reaching to the borderline of the horizon. There is an illumination, bright or dim, gay or gloomy. There are different regions, as Orient and Occident, and different areas, like home and abroad. Experienced space is not a mere object of perception; it is also not identical with so-called phenomenal space. Before we can hope to understand the pathological experience of space we have to enlarge and to correct our knowledge of the normal experience.

The same is true for time. Time is experienced not as so and so many minutes, but as a morning, an afternoon, or a day, a day with rain or with sunshine. It is not insignificant that in French the word “temps” means both time and weather. “Il fait beau temps” does not mean: “It is a beautiful time” but “The weather is good; it is a pretty day.” Likewise we do not refer to seconds, minutes, or hours when we say “We had a wonderful time.” When we speak about time we usually speak of manifold relations: earlier-later, before-after, one-after-the-other.

But – and this is the second difficulty in dealing with these problems – there are experiences where the temporal character is much more hidden. Not without some effort and skill can we thus free the temporal ‘elements’ from the compound in which they exist. For the exploration of the norm and the necessary material can be procured: we can rely on our own experience; we can communicate with others. In the study of pathological experience, however, we depend on the willingness and the capacity of patients to tell us what they experience. Sometimes they are willing to describe, but

incapable of describing, what has happened to them. Their experience is so new and strange that our everyday-life language fails to provide a satisfactory means of communication. The stronger the disorder in the experiencing of space and time – and thereby of all other experiences – the deeper the gap which separates the patient from the world we have in common. To point to a catatonic patient and to claim that in such a case the disorders of space and time are probably most severe seems like begging the question. We may be indulging in fictions and fancies; the patient will not refute them but neither will he confirm them. But if we can demonstrate the truth of certain hypotheses about the normal experiencing of space and time, we may reach a point where we will no longer depend on the patient's willingness to communicate or capacity for communication. In his behavior we may find an "objective" for deciphering the order of space and time which he experiences. This is a goal perhaps to be reached sometime in the future. We are fortunate at present if we find a patient who gives us an understandable description of his experience.

Clinical Case Study (Part I)

Such is the case of A. Br., which we may examine with the hope of confirming or refuting one possible hypothesis about the nature of the experiencing. A. Br., a teacher, was admitted to the Henry Phipps Clinic at the age of 45. During a state of depression, the second severe attack in his life, everything appeared big to him. Dr. M., the house officer, who first took care of him, looked like a giant, as if his size were eight feet, although the patient knew that it was less than six feet. Out of doors, the impression of enormity was even more emphasized and uniform. At the same time that things seemed to be big, they also appeared flat and far removed. They all were equally remote. There was no longer any differentiation between distances, between closeness and farness; spatial perspective was lost. The patient described his impression as comparable to the drawings of children or to those of beginners who have not yet learned how to represent perspective.

Owing to the lack of perspective, the patient felt at a loss when, in throwing a ball on the baseball field, he had to estimate the distance to an intended goal. The patient added that in former days he was a good baseball player and very sure in estimating

distances and in determining the right direction. But now all differences of distances were erased. There was no longer a possibility of articulating remoteness and of subdividing distances. One might say that distances with their quantitative differences of lengths had been swallowed up by the uniform physiognomy of remoteness.

Also the appearance of things in motion was strangely altered and distorted. A ball, for example, seemed to move from one position to the next one, similar to the sequence of frames in so-called “slow motion” photography. The disruption of the continuity of motion added to the frightening aspects of space which A. Br. complained about: the lack of resonance, the emptiness and deadly silence. These disturbances in the perception and performances of motion lasted for a long time. The resulting loss of spontaneous adaptation had already forced A. Br. to give up the driving of his car several months before he asked for admission. Several months thereafter, at a time when his conditions were considerably improved, he still encountered difficulties when he had to cross a street. Either he waited for a long while before he dared to cross, or he “just made it.” When he was riding in a bus, he had no feeling for the right moment to get up and to move towards the exit.

In his immediate environment in the ward, A. Br. was well oriented. However, there was a definite disorientation in regard to more distant goals. As a climactic point of his disorientation A. Br. reported the following event: he, in the fall of 1944 went to visit his brother, who lived in a small town in South Carolina. During this visit A. Br. once went to the post office. Although he had no difficulty in reaching the building, marked by the flag pole and flag, it took him hours to find his way home. He was too shy to ask anybody for help. He finally succeeded by trial and error.

Later on, during his stay at the Clinic, he had difficulties in finding his way around Baltimore, a city which was very familiar to him. He tried to overcome his embarrassment with the help of a map. A. Br. also reported experiences which belong in the realm of phobias. Before his admission he lived in a small town in the neighborhood of Baltimore and used to go for walks there. During these walks it happened several times that, at a point where the highway stretched into the open countryside, he could not go on any farther. He felt as if he were nailed to the ground.

For his way home, he chose the railroad tracks, where he stepped from one tie to the next. As this track was abandoned, one may discard any hypothesis (suspicion) that a tendency toward suicide was at work.

Things, although changed in their size and in their physiognomy, had not lost their familiar shape – with one exception. The clock in the ward appeared to be "not round" any more. A. Br. insisted that "not round" was the right expression. The impression was not that of a square, an oblong, or any regular or irregular polygon; it was just "not round." As the clock consisted of a round, white dial in an oblong, brown case, the alteration was in fact limited to the dial, i.e. to an object in which the orders of space and time converge. It is important to keep in mind that no other object underwent a similar distortion. A. Br. said that he knew perfectly well all the time that the clock was round. Nevertheless, he could not free himself from the frightening impression "not round"; he avoided most carefully ever looking at the clock.

About time, A. Br. gave an account which I will simply report here, leaving all comments on these paradoxical and seemingly contradictory statements to later, more systematic, discussion. A. Br. said "Time has ceased to exist." He could not remember the previous morning; it was as far away as events which had taken place years ago. All events were remote, and equally far away. There was a loss of temporal perspective. True, there still was a change of day and night. However, in spite of this change, there was no beginning and no end to things; time had become amorphous and continuous.

The precision of A. Br.'s utterances shows that he was never in a state of confusion. He was fully aware of the change which had taken place in him. The discrepancy between his immediate impression and his knowledge and the impotency to realize his knowledge in actual sensory experience added to his discomfort and pains. This split between sensing and knowing, between personal momentary experience and objective, lasting knowledge, is no unique phenomenon; it is not confined to disturbances in the experience of space and time. I may take up a discussion of it in a special paper.

Published as a supplementary material to:
M. Moskalewicz, T. Fuchs, Psychotic disorders of space and time – A contribution of Erwin W. Straus
Frontiers in Psychiatry 2023, https://doi.org/10.3389/fpsyt.2023.1150005

The Primacy of Physiognomic Characters

In the foregoing report, I made use of the terms physiognomy and physiognomic. Although Kurt Koffka and a few others have referred to physiognomic characters, this concept has not yet become a generally accepted part of psychiatric terminology. The term "physiognomy" – as I want it understood – encloses a good deal of a theory of sensory experience. A single definition, therefore, even if it were unambiguous, would still not be sufficient to make the full meaning of this term understandable. To start from an example serves our present purpose best.

Let us suppose, then, that someone goes for a hike in the mountains. At some point he misses the right trail. When night falls he finds himself in an impassable region of cliffs and rocks. He can move neither forward nor backward. Darkness forces him to remain for hours, almost motionless, at the same spot. Finally dawn announces the end of a very painful night. With daylight he discovers a small path not far away; he reaches it and is safe.

This man has experienced the physiognomies of night and day. For him they certainly were not groups of sense data. He came to understand what we all knew before we ever opened a textbook of psychology, namely, that our sense organs are not merely receivers of stimuli, that there are no sensations in our mind which we interpret as signs of the existence of an outer world, but that through our senses we are connected with the world, and that, in seeing, we are in a specific contact with the world. Our mountain-climber has realized that seeing is an organ, i.e. a tool, of motion. Night closed the world for him and daylight opened it up again. Visible impressions are related to us as potentially mobile, as potentially acting, beings. As potentially acting beings we experience the world as resistant or supporting. There is no sensory experience in which a power-relation is not involved. A being capable of action is at the same time such a one as reacts, suffers, is affected.

It is the ill fate of the theories of sensation that sensing has been interpreted – almost without exception – from the point of view of knowing. However in perception and in observation, especially in scientific observation, man has another attitude towards the world, towards himself, and towards his fellow men, than he has in

sensing.

The subordination of sensation to knowledge necessarily put an undue emphasis on the psychological inquiry into the "perception of space." During the protracted discussions between nativists and empiricists about how the idea of space is acquired, both parties tacitly agreed that the idea of space was the idea of *the* space, i.e. of geometrical, Euclidean space. On this basis (with this pre-supposition) they could ask whether space is perceived by all senses or only by some of them, and by which ones. This debate was carried on until recent years. It makes sense only if one supposes that the discussion is about *the* space. Obviously one cannot demonstrate Pythagorean theorem in acoustical space. But this is no reason for denying specialty to the acoustical appearance of the world. It appears to be a sufficient reason only because of the assumption that at least a possible transition from sensory space to geometrical space is to be required. When this requirement is not fulfilled, there space is not experienced.

The experimental analysis of the perception of space could not but accumulate more and more discrepancies between geometrical and sensory space. Ewald Hering's differentiation between phenomenal space (*Sehraum*) and objective (i.e. geometrical) space was an important step, not only toward accounting for the discrepancies, but also toward freeing the psychology of space from an epistemological dogma (Hering 1861-64, 1879). However, Hering's main interests and his methods were those of a physiologist. For him, too, space remained an object of perception. Hering's description and physiological analysis of spatial sensation did not lead him to a new concept of sensing, so that, while his work contributed to the loosening of the fetters, it did not break them. The decisive impetus comes from mathematics itself.

The development of mathematics since Nikolai Lobatschewski has not diminished the gap between the concepts of geometrical and phenomenal space. Quite the contrary: There is no longer any intuitive scheme of space; transition from sensory space to non-Euclidean space is no longer possible. But with the discovery or invention of the so-called meta-geometrical forms of space, the three-dimensional, homogeneous, isotropic space has lost its prerogative and consequently visible space has lost its preeminence. The space of old days became

nothing more than one possible scheme among a variety of others (Cassirer 1929).

Thus, the philosophy of space was confronted with new problems, but also with new possibilities. Psychology, followed at some distance by psychopathology, took notice only hesitantly and reluctantly of psychology, even though this development had opened a new avenue for an attack on the problem of expression. It is clear that, if perceived space need not be akin to geometrical space, things perceived in that space need not be akin to the objects of physics. Wolfgang Koehler from his observation of chimpanzees and Kurt Koffka from his observation of children came to the conclusion that features like "threatening" or "tempting" are more primitive and more elementary contents of perception than those we learn, as "elements," in the textbooks of psychology. In this respect, Koffka, following Max Scheler, Heinz Werner, and others, used the term "physiognomic character."

Koffka tried to explain the origin of physiognomy by a "hypothesis about the nature of the organization of the field." He assumed that physiognomic characters "arise in objects when these objects are in dynamic relation with the Ego, when, otherwise expressed, a state of tension exists between them and the Ego" (Koffka 1935, p. 360; Koffka 1928, p. 150).

I am afraid that the tendency to force all phenomena into the scheme "organization of the field" hinders rather than furthers our psychological understanding; Koffka's hypothesis does not explain why physiognomic characters are possible, and, still less, why they are necessary contents of sensory experience. The moment one takes the term "organization of the field" as more than a physicalistic analogy for experiencing one is losing sight of the phenomena themselves.

In the traditional interpretation of sensation as an element, the material, or the preliminary stage of perception, the subject of sensation becomes an observer who looks, so to speak, from outside into the world. He is interested in making statements *about* something, *about* events in the field under observation, *about* their mutual relations, or *about* their relation to a general frame of reference. It does not make much difference whether one refers to the observer as a mind, a consciousness, or an Ego. The observer as an individual being does not himself enter the scene; he is kept aloof from the events observed. Observers are

supposed to be inter-exchangeable.

The subject of sensing, however, is no Ego of this kind; it is a living being who exists within the world in a constant exchange with it, in absorbing and excreting, receiving and giving, uniting and separating, in breathing, eating, loving. The senses serve primarily the vital existence of living beings – preservation, protection, procreation. That the senses, especially seeing and hearing, also may serve the human desire to gain insight does not contradict the fact that they primarily serve vital existence. This statement does not imply an evaluation; "primarily" does not say "better"; it does not mean "first on the scale of values". Because I am trying to discriminate sensing from knowing, and to give to sensing what belongs to it, I want to emphasize my personal conviction that it is an excellency, the human excellency, to go beyond vital existence. The statement that senses primarily serve vital existence is no praise of the irrational forces.

Experiencing beings, men and animals alike, are directed towards the world as to the Other. Like insects, which stretch forth their antennas ahead of the locomotion of the whole body, we are, through our senses, directed to the world, ahead of ourselves. We not only are directed to the world as to the Other, we are also directed to another phase of our own existence, its preservation or annulment. If something attracts me, I am aware of it at this actual moment; but I experience my relation to it as not-yet-being-united. This peculiar temporal structure of attraction and repulsion and of every conative attitude is relatable only to the experiencing being as a whole; for this reason, sensation and mobility cannot be actually severed from each other. Because, as experiencing beings, we are ahead of ourselves, directed to the other, we find the world responding to our conation – we experience it as threatening or comforting, as resisting or agreeing; in other words, we necessarily experience physiognomic characters.

As I encounter the physiognomic characters, in so far as I face the world, the physiognomic characters are directed against me as against this concrete single individual; they affect me in my vital existence. This centripetal direction of sensory experience, frequently forgotten in our scientific attitudes, becomes most obvious in pathological cases.

The proportion s:t in the equations expressing the motion of falling bodies does

not concern me as this concrete, single individual. The less I am touched, the cooler and the better will my judgment be about the ratio s:t. However, in jumping into the cold swimming pool I am affected, not my Ego but I, as this individual, living being. In every sensory experience we also experience our body, our physical existence, in a specific perspective.

Physiognomic characters originate within my I-world relationship. Their specificity depends both on the object and on myself, also on such conditions as my strength or on my weakness, on my freshness or on my fatigue, on my indifference or on my sensitivity. Men can learn to separate physiognomic characters from the original I-world relation, to objectivate them and to represent them. The poet does it, the painter likewise; we all do it in our judgment of expression. Physiognomic characters carry the authority of evidence. In a conflict between the expression and the content of an utterance we will rely on the expression. Sensory physiognomic evidence and spontaneous reaction to expressions may lead to right judgments, even without preceding experience. But physiognomic evidence is no necessary criterion of truth. Deceptive evidence is one of the main topics in psychiatry; it prevails, for example, in ideas of reference, in hallucinations, and in some forms of delusion. Nevertheless, since physiognomic characters originate in an individual I-world relation, their experienced evidence has some validity. It indicates the actual relations existing at the time between this individual and his world. The individual who exists in that relationship experiences the physiognomic characters as unmistakable, as a verdict against which there is no appeal. To him they appear as objective data. For him they determine what something is or could be. The individual is overwhelmed by the power of the physiognomies. The psychiatric observer, therefore, can use the physiognomic characters described by the individual in order to judge the state of his mind. From the structure of experienced space in A. Br's case, for example, we may come to conclusions about the partner of this I-world relationship, i.e. about A. Br. himself.

Conversational language is in our days not coined for the expression of physiognomic characters. Old physiognomic terms grow obsolete and the young generation of words which makes its

entrance into our vocabulary clearly shows the mark of its origin in a machine age. Psychotic patients not infrequently lack adequate means for a communication of their experiences. Sometimes their modes of speech are close to poetical language, undoubtedly because their experience is dominated by physiognomic characters. At other times they use words of common usage with a definite physiognomic connotation. Their speech, therefore, may reach a high degree of ambiguity; we, the observers, may either fail to hear the physiognomic intentions, or we may read them into physiognomically neutral words.

Clinical Case Study (Part II)

"Big" was one of the words A. Br. preferred to use for a description of his spatial impressions. "Big" need not carry a physiognomic meaning. We call a house big, or a trunk, or a dog, if the size of an individual specimen surpasses that of the average in the same group of things. A. Br. was quite capable of pointing out that this was not the meaning of the word "big" for him. Dr. M, his physician, A. Br. said, was big, was a giant. Yet A. Br. was well aware that Dr. A., another physician with whom he came in contact, was taller than Dr. M. – still Dr. A. did not appear as big to him as Dr. M. When the patient compared the size of the two men, or when he related the size of each of them to an abstract means of measurement, the result did not coincide with the impression "big." In comparing one with the other, he acted as an observer. "Big", however, expressed a direct relation between him and another person or object; it was used as a physiognomic term. A. Br. said that Dr. M. appeared to him *as if* he measured eight feet, although he knew his size was less than six. The "as if" indicated that a word was used not in the common way, but with a peculiar, unusual connotation, a quantifying term in order to express physiognomic qualities. "As if" is a phrase which we encounter frequently in other cases of depersonalization, and for the same reasons. In looking at Dr. M., our patient felt as if he were in the presence of a giant – in other words, he himself felt like a dwarf, overwhelmed, overpowered. This power need not be hostile; it may be benevolent. However, "big," "flat," "remote" all these words used by A. Br. for a description of experienced distortion of space point to the same basic fact: that space had gained a different, frightening

physiognomy, because the power relations between the patient and the world had been changed. Under psychotic conditions these power relations may become more obvious; they do not, however, originate in or through the psychosis; they are a permanent outfit of all sensory experience. Under normal conditions, as long as we are capable of dealing with things and of moving freely, they may escape attention. They are more emphasized in psychoses because the change in the power relations is one cardinal trait of psychotic conditions.

One could come to the conclusion, by following A. Br's description, that the physiognomy of "everything" was changed in the same manner. Later on, it was learned that the term "everything" had to be taken with some reservation. In the immediate environment, especially, not everything suffered the same alteration. Forks, spoons, plates, the toothbrush, i.e. tools which A. Br. was still able to cope with, were not affected; but books and magazines, even as small in size as the "Reader's Digest," were affected, because the patient, unable to concentrate and really to absorb what he read, had no power over them.

Some psychiatrists have acquired the habit of referring all symptoms back to a supposed origin in childhood. However, A. Br.'s behavior contradicts the assumption of a plain revival of childhood experiences. Children have little power over their spoons and forks and they used to feel more at ease in handling their first picture books. Childhood experience can determine the actualization of potentialities – and thereby form character traits – but it does not produce character traits in them. Similarly, the father could not be experienced as an incarnation of power if the child did not experience the world from the very beginning as resisting and supporting, if sensory experience and the experience of power-relations were not indivisible. Physiognomic characters are an inseparable part of sensory experience. The first appearance of an experience in an individual's life does not mean its genesis at that moment, to say nothing of its being produced by the special condition prevailing at the moment of its appearance. The conditions prevailing in our early years may nevertheless decide to which physiognomies we will be more sensitive in later periods of our lives.

Bigness was only one expression of the patient's impuissance. He felt deprived of all power to deal with things; their size,

weight and pressure therefore outgrew all normal conditions. But there was another complaint – that everything appeared to be flat and remote, and that the perspective order of things was lost.

"Remote" will say that things are out of the patient's reach. In many religions we find the gods dwelling in a space inaccessible to normal beings. The Homeric gods lived on the height of Olympus. Jehovah called Moses up to Mount Sinai while the people were waiting down in the plain. From their heights the gods may reach men, but men cannot reach the gods. Their remoteness is an expression of their superior power. In Calvin's theology God is put at an infinite distance from the world, from earth and men. He is unapproachable for men. Neither work nor faith nor prayer can change God's will. Could men's prayer move God, man would influence God, an idea which completely contradicts Calvin's idea of divine omnipotence. The dogma of predestination followed from this view of God's inaccessible remoteness. *Finitum non est capax infiniti* – the finite is not capable of the infinite.

Bigness, remoteness, inaccessibility, are melted together in the majestic view of the Alps. Bigness alone may still permit closeness. With the giant we may still have contact. Even when we feel his overwhelming power, we still feel something. Remoteness throws us back into solitude.

A patient who complains of the remoteness of things tells us that he can no longer lay his hands on them, that he has lost all contact with them. Big and flat are qualities which could be misunderstood as attributes of things, noticed by a detached observer. Remoteness clearly signifies a relation between things and a person who can no longer reach them. Remoteness is a physiognomic character, experienced by someone only in so far as he intends unification. The juxtaposition of big, remote, and flat leaves no doubt that A. Br. with all these words pointed to physiognomic characters.

Flat things no longer have volume which permits them to be seized. They do not respond to our intentions. They appear remote and flat because the patient has become incapable of aiming at them. The space, the landscape, in which the patient lives is frozen, and torpid, and the colors are dull; there is a deadly silence. Sounds, Mr. Br. said, were "cutting through him." In this predominantly optic form of space, dancing

becomes impossible. So it was with A. Br. when he tried to participate in a dance; he found himself executing dance motions mechanically, but not drawn into the common rhythm.

Loss of Perspective

A patient whom Minkowski quotes expressed his experience in this way:

"There is nothing but immobility around me. Things present themselves in isolation, each one for itself singly, without evoking anything in me. Certain things which should constitute a remembrance, evoking numberless thoughts, giving an image, remain isolated. They are comprehended more than felt. It is as if a pantomime were performed around me, but I do not enter into the play. I have to stay outside. I still have my judgment but the instinct of life is gone. I do not succeed any longer in displaying my activity in a sufficiently lively manner. I cannot pass from minor chords to major ones; nevertheless, one is not built to live with one theme only. I have lost contact with all kinds of things. The conception of value, of the difficulty of things, is gone. There is no longer any current between them and me; I cannot abandon myself to them. Absolute rigidity surrounds me. I feel still less mobility toward the future than toward the present and the past. There is in me a kind of routine which does not permit me to envisage the future. The creative power in me has been abolished. I see the future as a repetition of the past" (Minkowski 1932-33, p. 236).

For the moment I still have to disregard the temporal phenomena; they will be discussed later. But it is important that in this report, also, spatial and temporal disorders appear side by side. What Minkowski's patient had to say about his experience of the distortion of space agrees completely with the complaint of our patient. I could quote other examples from English and German authors; the differences in language and cultural background do not prevent perfect conformity – a reliable sign of the basic nature of these disturbances. Descriptions, like the one just quoted, confront us with a definite type of pathological experience.

"I have lost contact with all kinds of things," says Minkowski's patient, a statement which corresponds to A. Br.'s statement that everything is remote. Because all things are remote and equally remote, the perspective order is lost. There is no longer a center from which the surrounding space is articulated. The differences of lengths of distance, which still might be seen, get lost in the qualitative uniformity of remoteness.

Space has a perspective order, not only because of optical conditions; it is articulated in zones of reach, according to our capacities. The constancy of size – a phenomenon much discussed in psychology

– is preserved within a certain reach; it changes by jumps when something approaches the borders of the near zone. In the frozen surrounding of our patients, the organization of space in zones of nearness and farness breaks down. The patients are cut off from the world; their psychological solitude is expressed through their physiognomic spatial isolation. No way leads from their Here to any There. Space is divided into two sections. An insuperable gap severs the patient from the distant region; he cannot reach Over There.

Clinical Case Study (Part III)

In this connection it seems worth mentioning that A. Br., after a series of electroshocks, was tormented by smells, which emanated from everything in his surroundings. Whatever their quality, it was unpleasant because of its obtrusiveness. While visible objects appear in a distance opposite to us, removed from us, so that we are directed towards them, odors and scents move towards us; they catch us and take hold of us. It seems to me that the obtrusiveness of smells in this case was not the immediate physiological effect of the electroshock therapy; the threshold had not been lowered. The obtrusiveness of smells was also a physiognomic phenomenon. Yet the direction was inverted, things were coming toward him, while he was not yet sufficiently improved to deal with them. The change of power-relations prevailing during the shock therapy and shortly afterwards found its physiognomic expression in the specifically fitting modality of senses: in the area of smelling.

With the physiognomic dismemberment of space, performance and perception of motion change accordingly. In a baseball game A. Br. was not able to aim correctly at a goal. When he looked at a ball in flight, he had an impression of discontinuity comparable to that of “slow motion.” Under normal conditions, points Over There are potential goals; the ‘‘there” lies ahead of me; I am moving towards it, as to a point which I will reach in the future. Space, visible space, unfolds itself and is open to the future. Space is not a timeless order of places side by side; it is a field of potential action. From me as its center, space stretches continuously into manifold directions. From my “Here” the other points around me are determined as “There,” as possible goals. The experienced space in which we live and move is relative to us as movable beings, as potential actors.

Movable beings mean beings who are in their actual situation, Here, potentially Over There; it means beings who, while living in the present moment, can anticipate the next one. A continuous group of centrifugal and centripetal directions binds the mere side-by-side together in an egocentric organization. When this organization suffers, then, the context is dissolved; visible space is frozen in timeless inflexibility. The "There" is experienced in disconnected remoteness. The patients cannot reach beyond themselves; they cannot anticipate a goal. One among my patients described this loss of egocentric organization and the corresponding dissolution of space by saying that he saw perfectly well the chairs and persons in his room but could not feel them around him.

Under normal conditions a ball thrown is seen as leaving a starting point and approaching a continuous move an anticipated goal. In the frozen world of A. Br. there is no anticipation of a goal and consequently no continuous motion towards it. A ball thrown is not seen as its way towards its goal but as passing through a series of many separate positions, i.e. it is seen as in "slow motion."

I failed to examine A. Br. with tests patterned after the experiments used in psychological laboratories. The omission is not, however, too serious; for during the first months of A. Br.'s disease such experiments might have proved impractical while in later months the results would have lost significance. In administering tests of that type, one has to face two different problems: first, whether a patient is capable of accepting the role of an observer, and second, if so, how far his performance agrees with or deviates from the average score. It seems not impossible that A. Br.'s judgments would have remained within the normal limits of errors as soon as he was capable of functioning at all as an observer, because physiognomies are neutralized in the process of observing. They have to be neutralized if observation is to be possible.

A. Br. himself made arrangements for the desired tests. He did it on his excursions downtown when he used a map in order to clear up his spatial confusion. While in a laboratory experiment the first condition is to abstract from oneself, his intention was to apply an abstract scheme to a concrete situation. For years A. Br. had been familiar with the geography of Baltimore; yet when he was allowed to leave for some errand he soon found himself

perplexed and unable to find his way. His main difficulty was in visualizing the *sequence* of streets well known to him. Under normal conditions we project our way from a fixed center, home or office, or from our temporary position. Through the anticipation of a goal the purely geometrical scheme becomes temporalized. In the chosen direction of the egocentric – in this special case, centrifugal – organization of space, streets are ordered in sequences, one after the other. In Br.'s disorientation the same disturbance as encountered before was at work. The map proved of little help and A. Br. soon gave up its use.

Lived Space-Time

Physiognomic characters are more than a mere varnish. The shape of objects of everyday use originates in a matrix of physiognomic relations. Physiognomies serve us as the first determinants of the essence of things. That someone looks cruel or mean tells us more about him than the fact that he is a teacher or a broker. Even the statement that a woman looks very feminine says something more significant than her belonging to the female sex. In many psychotic states this predominance of physiognomic characters becomes most obvious.

The experience of visible space, too, does not depend on optical, physical, or physiological factors alone; it does not depend on the functions of our sensory organs alone. It is likewise not sufficient to consider the influence of the vestibularis and of the frontal tone systems. There are, one might be inclined to say, emotional factors contributing to the unfolding of our phenomenal space. This statement may be correct; however, it does not say much, owing to the vagueness of the term "emotional factors." Although the foregoing analysis permits us to speak with somewhat greater precision about the emotional factors, a deeper insight depends on an understanding of the temporal phenomena.

The change of the physiognomy of space was, in A. Br.'s case, intimately related to a change of the physiognomy of time. But even if our patient himself had not directly reported strange temporal experiences, the analysis of his spatial disorders would force us to extend our considerations to time and its disorders as well.

As space, in our original experience, a field of and for action, the world is experienced as a living counterpart to our

own activity. In action we are directed to alteration. Whether it is simply a change of position or a transformation of objects into another state, or any interpersonal relation, we experience ourselves – it does not matter on what level of awareness – in transition from one phase of our existence to another one. Change, anticipation, transition, all indicate that the world has for us a temporo-spatial character. Space separated from time and detached from action is artificial. The constitution of the spaces of physics and mathematics is still more remote from our original experience of space, or, to be more correct, from our experience of ourselves in the world.

What does someone mean who, like A. Br. says: “There is no beginning and no end to things; I cannot remember this morning; time has ceased to exist”? What experiences does he intend to describe? How are these experiences possible? We have little doubt whether these are pathological phenomena. In other words, we take it for granted that normal experiences have been distorted and appears distorted. Obviously, we cannot hope to understand a pathological case without first inquiring into the norm of experiencing. Not only the explanation, but also the very observation and description of temporal disorders depend on a previous understanding and interpretation of time.

As was said earlier, we see only what we know. Introspection is not exempt from this limitation. In our daily work as psychiatrists, we order the phenomena observed in a single case into a prefabricated scheme of motions. When this scheme resists a smooth regimentation, we are perplexed. One way of escaping from such an unpleasant situation is to ignore the obstinate facts, but a better method of handling the situation is to take our embarrassment as a hint that the general scheme of notions needs a revision. Furthermore, apparently pure observation and plain description comprise a great deal of theory. Those who confine their interest to practical problems frequently find the volume of theoretical considerations necessary for the explanation of a single case out of proportion. Yet a single case might be important; the recent history of physics offers many striking examples – precisely because it demands a reconsideration and clarification of assumptions usually taken for granted. There is little danger of too long a digression; there is much more danger of confusing the issue by an attempt at condensation.

Published as a supplementary material to:
M. Moskalewicz, T. Fuchs, Psychotic disorders of space and time – A contribution of Erwin W. Straus
Frontiers in Psychiatry 2023, https://doi.org/10.3389/fpsyt.2023.1150005

Deficiencies of the Psychology of Time

The psychology of time, the heir of a long tradition, is tainted with some hereditary deficiencies. One of them is to reduce the experiencing of time to a perception of time. In doing this, psychology commits the original sin through which all that follows has been corrupted. With the reduction of the experience of time to the perception of time, the experiencing being is transformed into an observer. The observer himself, untouched by the flux of time, watches and simply registers in stoic calmness the sequence of external events. He notices their chronological order, the relations of earlier and later or simultaneity; he measures the lengths of temporal distances and compares them with his "subjective" impressions. For the observer, the clock assumes the role which geometrical space played in the psychology of space.

A good illustration of this scientific situation is to be found in the report of the Mayor's Committee on marihuana. Disturbances of space and time in Mescaline and Hashish intoxications are well known, and they did not escape the attention of the Committee's investigators.

> "If subjective criteria are of any value, it is interesting to note that many subjects reported that time passed very slowly for them when they were required to do something while under the influence of marihuana. In some instances, a performance which took only two minutes (for example, standing under an ataximeter) appeared to the subject to take a half an hour."

Nevertheless, all that the report has to say about temporal disorders is this: "Marihuana did not affect the ability of the subjects to estimate relatively short time intervals" (1944, p. 74-75).

Chronology and chronometry deal with only one aspect of time. It is true, our civilization is dominated by the chronometric order of time: by clocks and watches. If suddenly all clocks were to come to a standstill the gigantic technical apparatus would immediately collapse. However, even after this collapse we should continue to experience ourselves, each one of us his own individual existence in relation to the world, and – I may add – we should continue to experience ourselves in a state of becoming.

The perception-of-time psychology omits completely the fact that in every

experience we do not experience the world alone, but ourselves in relation to the world and in a continuously changing relation. The term “experience of time” is pleonastic; for there is no experience without temporal content and meaning. When we speak about the experience of time, we sever in abstraction for the sake of scientific inquiry that which in actual experience never occurs separately. It is only chronometric time that we can then turn our attention to or ignore. Perceived time is a content not necessarily present in all experience. But whenever we experience something it will be familiar or strange, transitory or lasting, finite or indefinite, for the first time or recurring, unique or repeatable, banal or important, boring or entertaining, a rehearsal or a performance. Chronometric time is conceived as an abstract, homogeneous, quantifiable extension. In primary experience time appears in meaningful characters related to our individual becoming, to our vital, social and different historical existences. Chronometric time is measured by seconds, minutes, hours; personal time is articulated by the values of the single moments in relation to our growth or decline. In perception of time experiments, time is identified with a measurable, general, common, objective order of one-after-the-other. Even if all results obtained are correct, they would say nothing about personal time, about the intrinsic temporal character of all experience. The method of inquiry is wrong in so far as it excludes the consideration of an important part – perhaps the most important part – of temporal phenomena.

Hume and James on Time

The identification of experience of time with the perception of time is not accidental. It is in full agreement with the assumption that there is but one science: mathematical physics, and that, therefore, its principles must also be applicable to mental life. Behaviorism and neo-positivism have shown how strong the idea still is that psychological experience should be ordered, described, and explained, in accordance with the “objective” methods of physics. The method determines the object. If the methods of physics were to be applicable, the object would have to enable their application. The object has to behave like an object of physics. It has to be measurable, quantifiable, countable. As we find a multitude of measurable things in the

physical world, thus, it would seem, a multitude of ideas, thoughts, and desires fill our consciousness one after the other.

"All the perceptions of the human mind resolve themselves into two distinct kinds, which I shall call Impressions and Ideas" (Hume 1874, p. 311). This apodictic statement of David Hume was repeated with slight variations 150 years later by William James. The phrasing of the opening sentences of his "Principles of Psychology" emphasizes still more this assimilation of mental life to the structure of physical things.

"Psychology is the Science of Mental Life, both of its phenomena and of their conditions. The phenomena are such things as we call feelings, desires, cognitions, reasoning, decisions and the like" (James 1931, p. 1).

One may argue that the plural: perceptions of mind, impressions, ideas, in Hume's terminology, and the corresponding plural: feelings, desires, cognitions, etc., in James' "Principles of Psychology" are harmless terms, unavoidable on the first pages of a psychological work. Yet these plurals imply a full-grown theory; perhaps it would be better to say their use is the expression of decisive theoretical assumptions. The presupposition is that mental life consists of a great number of separate, isolated, distinct parts. A theory of dissociation is at the bottom of all theories of association. It is this silent presupposition of dissociation which makes all understanding of the inner context of mental life possible. It is the same presupposition which introduces the clock time as the only temporal form in which mental life can be ordered; it prevents personal time, and becoming, and the relations of both orders of time, from even being noticed. James struggled against the assumption of a discontinuity of mental life. In the chapter about the "Stream of thoughts" he emphasizes the constant change of consciousness, which "is sensibly continuous."

"Consciousness, then, does not appear to itself chopped up in bits. Such words as "chain" or "train" do not describe it fitly as it presents itself in the first instance. It is nothing jointed; it flows. A "river" or a "stream" are the metaphors by which it is most naturally described" (James 1931, p. 239).

In the "Pluralistic Universe" James refers to Henri Bergson and to his view of the devenir, the becoming: "What really exists is not things made but things in the making".

(James 1909, p. 263). Yet, as James's discussion of time and his theory of emotions show, he could not free himself completely from the power of spatial motions. In the "Pragmatism" he turned back to Hume.

Hume, himself, was fully aware of the difficulties which grew out of his basic assumptions. I quote Hume because he is the classic representative of psychological atomism, because his ideas are still powerful today, and because his skeptic resignation leads directly to the exact point where the basic change has to be made. If impressions and ideas are separate from each other, if one thought enters the stage of consciousness when the other has left it, if there is a discontinuous sequence of indivisible parts, how can we experience time? Hume's answer is that "our ideas of space and time are compounded of parts, which are indivisible" (Hume 1874, p. 344). The parts, united in a compound, do not form a continuum. Hume defines their relationship as contiguity; he denies an immediate experience of time.

"The idea of time is not derived from a particular impression mixed up with others, and plainly distinguishable from them; but arises altogether from the manner, in which impressions appear to the mind, without making one of the number. Five notes played on a flute give us the impression and idea of time; though time be not a sixth impression... which the mind by reflection find in itself... it only takes notice of the manner, in which the different sounds make their appearance, and that (manner) it may afterwards consider without considering these particular sounds, but may conjoin it with any other object" (Hume 1874, p. 343).

Time, one sees from this quotation, is, according to Hume, not experienced immediately. It is noticed by hindsight. Yet, if the original impressions are timeless, indivisible, separate parts, and if all ideas are derived from impressions, the objective manner of their appearance cannot be realized by an experiencing being who is limited to the awareness of one of the impressions after the other and of one to the exclusion of the other. Hume himself insisted that "no connections among distant existence are ever discoverable by human understanding" (Hume 1874, p. 559). He spoke about the inconceivable rapidity with which different perceptions succeed each other. However, while this explanation might deceive others, it did not deceive Hume. Reviewing the insoluble problems originating from his theory, he feels forced to skeptic resignation: "For my part, I must

plead the privilege of a sceptic, and confess that this difficulty is too hard for my understanding" (Hume 1874, p. 559).

There can be no doubt that the awareness of different impressions and of their change and of the inconceivable rapidity of their change presupposes the experience of time. The "objective" sequence of impressions, for example "five notes played on a flute," cannot give the "subjective" impression "sequence," if one impression follows the other, each one as a single and separate thing, as an indivisible part. It cannot give the impression of "sequence," if the impression *of* a single tone is a single impression. It does give the impression *of* sequence, if the single tone is experienced as in transition from the "Not yet" through the actual present, the "Now," to the "No longer." When we hear a single tone and really hear it as a single one, i.e. as one *only*, we already have related it to potential other ones. To hear a single tone as one means to hear it in its limits, beginning and ending, and thereby related to, and differentiated from, the background of silence, and separated from other tones. The experience of oneness, as such, presupposes the possibility of more than one. We are aware of different impressions in so far as we can relate one to the other. If the assumption that the impression of one tone is one impression were true, consequently were confined to only one *pro rata temporis*, we could never be aware of this difference or of change. Yet we do hear a sequence of five tones as a sequence; we hear a sequence of many words forming one sentence. When someone speaks we hear the words from the beginning of his speech as parts which complete each other to make a full sentence. We hear sentences in their state of becoming completed. We hear them as capable of, and in need of, completion from the first word on. The first word would not be the first if not related, before they appear, to those which may follow. We realize, therefore, that a sentence has remained incomplete, has taken a surprising turn, or has been grammatically incorrect. In all this we experience the "single impression" in transition; in other words, we experience in so far as we are beings in a state of becoming and we experience ourselves in the world in a state of becoming. The single moment is never experienced in complete isolation but as a phase of our becoming. As a phase of our becoming the single moment is incomplete, and therefore related to other ones, to those which precede and follow

within the sensory horizon, and to the whole of our vital, social, and historical existences.

Temporality of emotions

In our emotions we experience the tension between the actuality of the present moment and the potentialities of our existence. Indeed, it is this temporal relationship A:P which determines our emotional states. The actual moment gains its meaning and importance, even its specific content only in relation to the future and to the past, and to the permanent structures and to the virtual whole of our lives. Past and future are someway present in the actual moment.

We enjoy living on the spur of the moment, submerging ourselves in the present, forgetting about past and future. This forgetfulness is one condition of our euphoric states. As this forgetfulness is artificial, the euphoria is inevitably followed by a hangover, disappointment, or some sort of sadness, when, with the return of soberness, past and future, responsibility and consequences, also return, and when the actual moment presents itself as a phase shaped only by its relations to the virtual whole. When in the quietude of our visible horizon something appears that signifies potential danger, the meaning and emotional content of the present moment is suddenly and immediately changed. The future stretches forward into the present. We live between the two poles of preservation and annihilation; in every single moment we have a definite position between these two poles. With the appearance of something threatening we are moved closer to the pole of annihilation. We experience this change, this shortening of the distance in the direction of annihilation, as fear. For all practical purposes it might be sufficient to say that from a certain moment on we were in fear and at another moment we stopped being afraid. But we should not forget that in the emotion of fear we realize the character of the actual situation only in relation to the permanent background of safety and danger (preservation and annihilation).

In the context of our becoming, i.e. in the history of our lives, every moment has a unique place value. The categories, new-old, familiar-surprising, banal-touching, are applicable only to the history of an individual or a group. The historical significance of a single moment depends on the events in our environment but not less on our own capacity to integrate these external

events into our personal histories and to relate the actual moment to our past and future. For the man who, sentenced to death, watches in the solitude of his cell with growing horror the approach of his last hour, the sun does not shine. To be sure, the astronomical phenomenon is a matter of fact; but its occurrence in the world does not become an event in the prisoner's life.

The Unity of Personal and Objectified Time

About actual events we always say that they occur now. "Now" is a personal adverb of time; it is – so to speak – always my, your, his, her, our now. The word "now" makes sense only in relation to the speaker and to his listener in the actuality of their conversation. If we sever *now* from the actual present and actual presence it becomes meaningless. *Now* is meaningful only in the context of my or our individual state of becoming. There alone it has its legitimate place, but there it is also indispensable.

In preparation for D-Day, orders were given in terms of clock and calendar. Every single action was scheduled in the general, common, objectifiable order of clock-time. But when it came to the performance of these orders the local commanders of small units did not and could not use such terms but would tell their men to do now this and now that.

Suppose someone has the order to wait at a certain point until 5:00 p.m. and then to advance in the direction of X-Village. When his watch tells him that the right moment has come, he will say: "Now it's five o'clock, now let's go." We have to realize the objective order of time in the actual moment; we have to integrate it into our personal history. Every moment of our experience is related to the two different orders of time, the common, general order of time which finally can be measured by the clock and to the individual order of my becoming. When I ask the question: "What time is it now?" I relate my personal existence to an all-embracing, common, general order; I experience myself in relation to the world. In a very instructive paper entitled "Experiential and Existential Time", Mueller uses the scholastic term the aevum. "If many existential time structures are correlated, we have a historical time or age, the aevum" (Mueller 1946). Mueller quotes from St. Thomas' *Summa* the following sentences:

"Spiritual creatures as regards their affections and intellections, in which there exists succession, are measured by time; as regards their natural being, they are measured by the aevum; and as regards their vision of glory, they participate in eternity" (Mueller 1946).

The term natural in this passage refers to human nature as socio-political.

The general order of time is determined by impersonal or super personal astronomical constellations or by events in the world history: the foundation of the City in the Roman Calendar, the "fulfillment of time" in the Christian calendar, the Creation of the world in the Jewish calendar, in which an absolute beginning, a beginning of time itself, is posited. Everywhere we find the same phenomenon: the individual order of time, my becoming ordered into a common, general, embracing order of time, personal time related to world time; but everywhere world time also has to be reintegrated into personal history.

We assume that the time measured by the clock flows uniformly. We assume that 10 minutes today and 10 minutes yesterday or tomorrow indicate equal lengths of time. It seems to us that the extension of a year does not change whether it carries the number 1946 or 1945 or 1545. Yet in our experience on one day time passes quickly, in a trice, and on other days, the hours linger. Sometimes we wish that we could arrest and hold the current of time and sometimes we try, with equal futility, to push it forward. We know that weeks extend over the same period of seven days. Yet, looking backward, there is a perspective shortening of temporal distance; we are able to discriminate the seven days of last week but further backward the borders vanish as if the days were interpenetrating each other. With growing age, days, weeks, months, and finally years, appear shorter and shorter. In our fifties a year no longer has the length it had when we were high school boys. The biographical importance of events is the determining factor. The same external constellation lose weight in proportion of the experiences we have already gone through. The same constellation induces less and less decisive steps in our development, until with old age, when our potentialities have been consumed, life becomes more and more monotonous, our interests narrow down, and our attention turns back to the past. Nothing new can happen to us. If it were the number of impressions which determines our temporal experience a year in our fifties

Published as a supplementary material to:
M. Moskalewicz, T. Fuchs, Psychotic disorders of space and time – A contribution of Erwin W. Straus
Frontiers in Psychiatry 2023, https://doi.org/10.3389/fpsyt.2023.1150005

need not be shorter than in our twenties. Yet, because we experience in the context of our becoming and the importance of events alters in the course of our history, our experience of time undergoes a proportionate change resembling the data of Weber-Fechner's law. Because the importance of events is decisive, time passes slowly on a boring day, but the day is short in retrospection, while on a day filled with important and meaningful events time runs quickly but in retrospection it was a long period we went through between morning and night. With events like the attack on Pearl Harbor all the past is suddenly removed to a greater distance. In all these situations there is some degree of concordance or discordance between the two orders of time, but never a full correspondence or agreement. We may awake by the clock, begin and end our work on the dot; we may feel hungry by the clock and go to bed by the clock; the individual may act like an automaton; personal existence may disappear in the process of mechanization. But in just such a meticulous, slavish obedience to the flock we feel more discordance than agreement between world time and our personal time of becoming.

Pathological Discordance Between Personal and Objectified Time

In pathological states the discordance reaches a maximum. Because time is usually experienced more in concrete characters than in the abstract order of chronometry, we should not expect a patient to describe his experience of discordance in chronological or chronometrical terms. When it happens that a patient is aware of the discordance, as A. Br. was, we may hope to see how the experience of discordance is related to an altered state of becoming, and, along with it, to a change in the proportion of A:P and to a change of the patient's emotional life. By considering the structure of the norm of temporal experience we are able to predict some possible disturbances. Whether they actually occur we have to learn from clinical observation.

A change in his state of becoming, a discordance between the common and the personal order of time, made A. Br. say that "time has ceased to exist." It is true that in these words there is no reference to two orders of time; A. Br. did not say "world time goes on flowing while my personal becoming is retarded"; but that is what he meant. How do I know that I am not reading

my own interpretation into A. Br.'s statement? First, from inference, and then, which is more important, from comments A. Br. himself made. The inference is very simple. A. Br. spoke about the cessation of time, although he was aware of the change of day and night, and mentioned, for example, the remoteness of the same morning. In spite of his strange and bewildering experience, his thoughts were not incoherent. He could grasp the obvious contradiction between his statement that time had ceased to exist and his reference to the same morning. The conclusion that A. Br. could not have meant anything else but the personal order of time was confirmed by the patient himself. A. Br., when confronted with his contradictory statements, not only immediately saw the contradiction, but also gave the clue for its explanation: "there is no more advancing towards the future."

The anticipation of the future, the advancing towards it, are phenomena related to the personal time of becoming, either of one individual or of a group. My personal now, my actual present, separates the past from the future. In physics two events may have the relation of earlier and later. This relationship is not identical with that of past and future. The atomic bomb test at Bikini will first occur and the reports will follow it. Both events belong at this moment to the future. The invasion of Africa preceded J-A day, but both events belong now to the past. Next Tuesday the Bikini experiment will belong to the past. The reports, however, will be published later; and some months later they also will belong to the past. While the relation earlier-later among these groups of events is unchangeable, their temporal character may change. That which today belonged to the future, tomorrow will belong to the past; today will be yesterday tomorrow. In the time of physics the distinction between past and future has no proper place. A physicist, speaking about a future event, relates this event not to another physical event but to himself, to his actual present as an observer. The attributes past and future are always related to the speaker or writer, to his actual and constantly changing present. Past and future are related to an experiencing being and to the actuality of his becoming, to his history *in statu nascendi*. Someone who, like our patient, feels that there is no advance to the future, that this cessation has annihilated the existence of time, speaks about his personal becoming.

Minkowski's patient, whom I quoted

earlier, felt that there was a kind of routine which did not permit him to envisage the future. "Absolute rigidity surrounds me. I feel still less mobility towards the future than towards the present and the past." (Minkowski 1932-33, p. 236). In Mayer-Gross's paper on "Depersonalization" and in Aubrey Lewis's report "The Experience of Time in Mental Disorder" many other examples are to be found. One of Lewis's patients said:

"The future to me is remote. I feel hopeless.... Before I could look to the future, but I can't now. There's something won't let Me….Time just doesn't appeal to me…. Time doesn't seem to go at all…. The whole day seems exactly the same. If I look at the clock, it doesn't mean anything to me at all" (Lewis 1932).

And Mayer-Gross reports:

"I can't explain, all is timeless, unchanging, hopeless…. I had no feeling of time at all – time was nothing to me…. Time just passes, I don't see any future…. Yesterday seems years ago. I cannot picture any future" (Mayer-Gross 1935).

With this blackout of the future, the experience of the present is changed. The present comes to a standstill. Nothing happens in the personal history, whatever may occur in the world. In these states world time cannot be integrated into personal time. If it is no longer possible to absorb the common order to time into the actual Now, which is always my Now, world time will lose, in my experience, its character of reality.

The scientific concept of reality and the primary experience of reality are very different; yet they do not contradict each other, nor do they exclude each other. In scientific meditation we determine the reality of things precisely through an act of severance; we divorce them from our individual existence. The reality of events seems established if they fit into an impersonal permanent order of the laws of nature. In my primary experience reality manifests itself in the actual co-existence of the world and myself. Those things are real which affect me; they must be related to my being, i.e. my becoming, and I to them. Neither lawful order nor the mere being-at-hand gives to things the sensory character of reality. This is to be seen in all cases of depersonalization. A. Br.'s statement that time has ceased to exist may, therefore, in the last analysis, also refer to world time. Its character of reality is abolished in spite of the persistence of change.

In some pathological cases the discordance between world time and personal time becomes a still more manifest theme of experience. When under normal conditions, we look down from our window on a holiday crowd in the street, then we may enjoy our rest the deeper when we contrast it with all the activity going on around us. All this is no affair of ours; close as it is, it is far away; its reality dwindles in its psychological distance. Yet there is a limit. Rest is no standstill. We enjoy rest as a transition from having-done to going-to-do; it is a suspense of activity which gains its actual meaning in relation to past and future. Thus also time, while it hastens away, regenerates and grows with every moment. In the standstill of becoming, however, the patient experiences time only as passing, vanishing, dwindling, dying away.

This is well illustrated by the following quotations from an interesting observation reported by Viktor Emil vom Gebsattel:

"I have to think continuously that time passes. While I am speaking to you just now, I think with every word: 'gone, gone, gone.' I am permanently in a rush. If I hear a bird chirping I am forced to think 'Now again a second passed and again another one.' The same when I hear the clock ticking. I have hidden my watch. But the watches worn by other people also disturb me terribly. When my sister writes she will come to see me next Sunday at 8:15 a.m., I cannot understand her; I cannot understand that people make plans and connect a meaning with the dates they set and yet remain calm. Therefore I feel so estranged from all other people, as if I did not belong to them, as if I were completely different. Also when I see people moving I am forced to think with every single one of their motions: 'one second, again one'... I frequently think that I have gained a new insight, not yet shared by others, but completely logical. I do not understand how one can see otherwise. Conditions are permanently growing worse.... The fractions of time to which I have to give my attention are growing shorter and shorter. It is connected with noises, with perceptions, and also with ideas of suicide because the whole is so weird and unbearable. With everything I am doing the distance which separates me from death becomes shorter. Yet I am not afraid of death; I think it is very beautiful; nevertheless the idea that everything perishes and that life shrinks continuously is dreadful. While knitting the emphasis is not that the knitting which I do grows, but that through its growth the span of life is getting shorter and shorter" (Von Gebsattel 1928).

Disconnection of Past and Present

Just as in the standstill of becoming the world time cannot be integrated into the personal Now, the past also is disconnected, severed from the actual present. Present is no longer experienced as the continuation of

the past. A. Br., who claimed he could not remember the same morning, meant that according to his own explanation the morning no longer belonged to the same day. Lewis' patient, whom I quoted before, mentioned a similar experience:

"I want to get something back to my mind that seems to have gone, to let me see the present and the future.... everything seems ages ago. Even getting up in the morning, I feel I've forgotten it; it seems ages ago" (Lewis 1932).

One of Mayer-Gross's patients used almost the same words:

"Everything I have done seems such a long time ago. When the evening-time comes and I think back over the day, it seems years away" (Mayer-Gross 1935).

A. Br. also felt that the same morning was no less remote than events which had occurred two or five years before. He knew, of course, that objective distances (periods) of a half day and of two years are different. But he could not realize this difference; he could not make it his own because his personal time was deprived of a meaning which would have connected the present more closely to a certain point of the past than with another. In their severance from the present all past events appeared remote, so far remote that the perspective of time was lost.

Indeed, in these cases time is void of all articulation. One day is not distinguished from the other, as is a holiday from a weekday. One of Lewis' patients had complaints very similar to those of A. Br.:

"There is no break in time. I'm passing through time; there is no day and no night. There's nothing divided between my getting up and my coming here and going back. It's all joined into one" (Lewis 1932).

Under normal conditions we articulate our days by our work or our merriments; we begin and end; we plan and accomplish; we anticipate and look backward. In this turning from one more or less meaningful event to another we create caesurae without which time flows amorphously. In the emptiness of a standstill of becoming there are no more accents, no thesis, no articulations which could join some hours together into one group, of a forenoon, of a day, and thereby separate from other groups. "There is no beginning and no end to things," complained A. Br., confirming through his sad experience the fact that the beginning and end of things are marked by hiatuses, by bar lines in the score

of our personal history.

The temporal disturbances in A. Br.'s case becomes transparent in their structure when we relate them to the true time order of experiencing, to a state of becoming. That the alteration of time, of becoming, also changes the physiognomy of space has already been mentioned in the first sections of this paper. There we saw how, with the standstill of becoming, space and things in space grew torpid, how the incapacity for anticipation cuts off the "over there" from the "here" and lets things be drawn to a remote distance, and finally how motion is affected by these changes.

"Looking to the horizon, I saw everything dead still, as if I were looking in fairyland.... The world looks perfectly still, like a postcard. It is standing still; there is no point in it. A bus moves along without purpose; it does not feel real.... Everything in vision (sight) is dead; the branches of the trees are swaying without purpose.... Everything is flat, stiff, artificial, altered" (Mayer-Gross 1935, p. 110-111).

These are the same patients who complain that they have lost their sense of time, that they cannot picture the future, that they feel that time is standing still, that all is joined into one.

The striking similarity between cases of patients differing widely in race, creed, and color seems to me a sufficient proof that we are confronted not with an accidental conglomeration of strange symptoms, but with an exemplary case, a syndrome, presenting single symptoms which form parts of a meaningful context. If we first have understood the norm, the syndrome is also understandable, viz., its deviation from the norm, its many details pointing to one basic disturbance, *trouble générateur* (Minkowski 1933, p. 211).

Normal and Pathological Anticipations of the Future

I have tried to interpret the manifold disorders of space and time in A. Br.'s case as manifestations of one basic pathological process, namely, of a change of the state of becoming. As A. Br.'s case and similar cases are to be classified as depressive reactions, one may well consider the possibility that the same *trouble générateur* is responsible for the totality of phenomena of at least one group of depressions. It will therefore be our next task to see whether other characteristic symptoms of depressive psychoses may likewise be explicable as manifestations of a disturbance of becoming.

I will use the same method as before,

assuming that a better understanding of the norm will provide for a deeper insight into pathological processes. Let me take up again the subject of our being, under natural conditions, directed toward the future and of our anticipation of the future – our specific expectations of future events determining our mental experience. "*Le present est grosse de l'avenir*," according to a formulation by Leibnitz. Expectation of the future does not necessarily mean that one is busy figuring out what will happen tomorrow, whether, for example, the President will veto the OPA bill, whether prices will rise or not. Expectation has many forms and much more subtle ones than direct specified anticipation of an event in its details. Limitation or boundlessness, confidence or diffidence, growth or decline, are such unthematic anticipations of the future.

I am writing these paragraphs on a Sunday afternoon. I have many hours at my disposal. Nevertheless, there is a limit – I have accepted an invitation for the evening. This limitation of my time, of my freedom to work as long as I want and to stop whenever I want, influences the mood and tension of this actual moment. It is of great importance whether the future lies wide open ahead of me, in an indefinite extension, or whether it is coerced by limitations of many kinds. An expiring appointment could be one, lack of funds another. A well-filled purse opens the future, and the converse is also true: a primary blocking of the future may be experienced as impoverishment, and, as we see it sometimes in depression, as poverty, a loss of property (the German language has the significant expression "das Vermoegen" – property, a noun, derived from the verb "vermoegen" – to be capable of). Confidence in the solidity of the world is another constant factor in our expectation. We expect, without giving precise thought to it, that our house will not collapse, the atmosphere dissolve, or this pencil vanish in a chain reaction. Things are familiar as long as they behave in accordance with our expectation. We are surprised if they turn out against it. We realize confidence as a permanent factor of experience the moment conditions make us suspicious. Again, it seems highly plausible that with a blocking of the future a familiar surrounding will appear strange, suspect, ominous.

In our expectation we have, under normal conditions confidence also in ourselves. "Confident" means that we will be able to meet demands, that we can trust in

our capacity to do the right thing at the right moment. In sleep and in going to sleep we turn away from the world, from our future and our past; we demobilize our vigilance. But we can only accomplish it if we have some assurance that the past is in order and have some confidence that things will go on, that we will awake and awake at the right time. If we cannot rely on ourselves, we are afraid of failure, we try to avoid failure by detailed anticipation and by careful preparations, through which we hope to eliminate the unforeseen. Hypochondriac reactions are in many cases an expression of a lack of self-reliance, a lack of confidence that our organs will function in the proper way at a given moment. We know, especially from sexual neuroses, how the lack of confidence, the anticipation of failure, influences the expectation of the future and determines the actual behavior. In the depressions of the later years we find hypochondriac ideas pushed to the point of nihilistic delusions. The awareness of the gradual decline of the physical capacities in elderly people is brought to an extreme. The blocking of the future is experienced as a complete failure, even of elemental functions, of breathing, eating, digesting. The absolute diffidence in the functioning of vital organs, the not-being-able-any-longer, is expressed as a not-existing-any-more of their stomach, bowels, bladder. In younger patients depressive states may be disguised by the phobias of cancer, i.e. by the fear of an incurable disease which leaves no hope, and with which the end is already virtually present.

In our expectation of the future we anticipate the next day not simply as a substitute for, and follower of, the present one. The next day or year is the meaningful continuation and expansion of our personal history. We experience the future as well as the present and the past in the context of our becoming. It is no accident that the term “growing” is used as a synonym of becoming. Growth is the criterion of becoming. In fact, we experience our life as growing, either in the proper sense of the word, or – in its deficient modes – as shrinking, as recapitulating, or as standing still. Together with the overturn from growth to decline and standstill, the emphasis is shifted from the future to the past, the past gaining more and more in weight. Some depressive patients experience the future as a mere recapitulation of the past. They sometimes try to push forward their standing time by mechanized actions,

knitting, weaving, etc., usually in vain. Even if they accomplish something, they do not advance with the growth of their work. They are fixed to the spot; they move as a treadmill. The balance of the past is never completely settled. We liquidate the past in advancing to the future. If the future is blocked we are thrown back to the past; we are forced to recapitulate and to repeat, to circle around the same point; this retardation is manifest in depressive brooding and in ruminating, those forms of obsession peculiar to melancholic states.

The Entanglement of Past and Future

The present is pregnant with the future; it has also been engendered by the past. Our past continues in our present, determines it and forms it. While the future is the realm of potentialities, not yet realized, undetermined, the past is the area of facts, defined and determined, fixed and rigid. While the future is the area of possible goals, of desires, of hope and despair, the past is the area of our accomplishments and failures, of satisfaction or regret and disappointment. Guilt ties us to the past and blocks the advancing to the future; but some pious resolutions for the future suffice a drunkard easily to dismiss his misdeeds. Our view of the past depends on our visualization of the future. When we look back to our past on a good day, we will be pleased to register our accomplishments, but on a bad day, when our progress to the future is blocked, we will be haunted by the number and stupidity of our mistakes. The past is a fact, in the etymological sense of the word: it is *factum*, something that has happened and therefore is unchangeable and irrevocable. Yet is not an impersonal, causal factor; it is a fact in our personal histories and it depends on the progress of our histories whether we will experience the past as a territory which we left behind us, as a solid ground which supports us, or as a trap which chains us. The past is illuminated from the present; in the changing light of our becoming the aspects of the past vary, although its basic character persists through all the variations. The past, as determination, is always a limitation of our potentialities. Whenever we act, whenever we decide, we give up possibilities. Every decision involved resignation. There is no escape from this necessity. Unavoidably, therefore, the past is also the score of our missed opportunities. We always are debtors to the past. Confronted with our past we are at the

bar of justice and there is nobody who can plead "not guilty."

The depressive patient is completely overwhelmed by the impact of the past. The alteration of becoming, the blocking of the future, turns him back to the past, which pronounces a terrible judgment from which there is no appeal. In depressive delusion history is experienced in its absolute irrevocability, the past as unpardonable guilt, the future as inevitable catastrophe, the present as irreparable ruin. Whatever the words the patient uses to describe his situation, whether he sees in himself the Antichrist or simply a sinner or a man through whose guilt the whole town will perish, he faces ultimate retribution, irrevocable chastisement, eternal punishment. In depressive delusion, in guilt and retribution there is an intrinsic temporal character, not of the clock time, but of the time of personal history; these are phenomena related to alterations of becoming, which, in some cases, probably are of biological origin.

I-World Relations

It has some theoretical and practical importance for psychiatry in general to understand how basic formal disturbances shape and determine specific attitudes. In this connection two problems still demand at least a short discussion.

Patients in severe states of depression fall, like other psychotics, into an impenetrable isolation. In the fragmentation of space, the remoteness of the "There," the inability to aim, the cessation of time, A. Br. began to realize a progressive isolation. He became incapable of joining a common motion; personal contacts broke up one after the other. A mute, inflexible side-by-side does not constitute a field for interpersonal relations. Whether friendly or hostile, they depend on the possibility of common action. The very disturbance in A. Br.'s case and in similar cases points to a problem more or less ignored, in spite of its importance. The question is: how is communication, how is common action psychologically possible? It obviously presupposes a freedom of motion, an exchangeability of the "Here" and the "There" and thereby of your and my position. Our personal relations rest on an attitude towards the world. All I-You or I-He relations, all We-formations, are specifications of our I-World relations.

Gebsattel's patient, being herself cut off from advancing to the future, complained that she could no longer understand other

people. She could not understand their anticipating the future or making plans of any kind. This difficulty in understanding others is the first step towards isolation. The patient feels himself excluded from the community or his fellowmen; they are strange to him. First he cannot understand others; next he will envy them, and then he will hate them. Aggression, rebellion, and delusions of persecution may have their origin in the loss of the world in common. There are definite conditions necessary for the possibility of positive interpersonal relations, only if I can move freely in the world, only if I can turn towards this or that goal can I meet – so to speak – the other one as my partner on the same journey. Interpersonal relations presuppose something which I can share with someone else. If I cannot act, the other one cannot be my partner, my co-actor. Interpersonal relationships depend on the possibilities of my free behaving in the world and on the possibilities of my conducting my own history. When, in a standstill of becoming, I cannot any longer step out from my personal time into a universal, common order of time, when I am cut off from acting in the world, I am excluded, simultaneously, from community with others. In the group of disturbances arising from a conflict situation the potentialities are preserved; their actualization alone is suspended; in the other group the potentialities themselves are afflicted. It seems equally important for the theory of psychoses and for psychiatric practice that we can conceive of disturbances which do not originate within the realm of interpersonal relations, but affect them, nevertheless, in a characteristic manner. Their existence, of course, does not contradict or exclude these which at present absorb almost all attention: the disturbances originating in conflict situations.

The Clock Illusion

The last point I want to mention concerns A. Br.’s frightening impression that the clock in the ward was no longer round, an impression from which he could not free himself, although he knew that it was illusory. It is noteworthy that the clock, and the clock alone, suffered from such a hallucination-like distortion. This was probably no accident; for in other cases also the clock alone was affected. Kloos (1938) reported on a depressive patient who at the climax of her psychosis noticed to her great amazement that the hands of the clock moved backward. We may, therefore, not be

wrong in assuming that "not round" in A. Br.'s words meant that, as time had ceased to exist, the hands were not circling any more. If this assumption is correct, we can witness in A. Br.'s observation a hallucination *in statu nascendi,* a physiognomic change, an alteration in the I-world relation, conditioning a change in the appearance of objects. Our observation of depressive patients may thus give us a clue as to how to approach the general problem of hallucinations. The elaboration of this hypothesis, however, does not lie within the scope of this paper.

My first publication concerning the changes of time in depressions was based on an analysis of the intrinsic temporal phenomena alone. I could not at that time refer to a case like that of A. Br., where the patient was aware of temporal disorders. A number of observations, published since 1928, have shown that an analysis of the formal structure of experiencing even enables us to predict the possibility of certain symptoms and their specific content. The actual occurrence of such symptoms can be taken an impressive confirmation of the theoretical view.

References

Binswanger, L. (1933). Das Raumproblem in der Psychopatholgie. *Zeitschrift fuer die ges. Neurologie u. Psychiatrie*, 145, 598-647.

Binswanger, L. (1942). *Grundformen und Erkenntnis des menschlichen Daseins*. Zurich: Max Michans Verlag.

Cassirer, E. (1929). *Philosophie der symbolischen Formen* (Vol. 3). Berlin: Brunne Cassirer Verlag.

von Gebsattel, V. E. (1928). Zeitbezogenes Zwangsdenken in der Melancholie. *Der Nervenarzt*, 1, 275-287.

Hering, E. (1861-64). *Beiträge zur Physiologie* (Vols. 1-5). Leipzig: Engelmann.

Hering, E. (1879). *Der Raumsinn und die Bewegungen des Auges. Hermann's Handbuch der Physiologie*, 3, Leipzig

Hume, D. (1874). *The Philosophical Works of David Hume* (Vol. 1) (T.H. Green and T.H. Grose, Ed.). London Longman, Green.

James, W. (1909). *A Pluralistic Universe: Hibbert Lectures at Manchester College on the Present Situation in Philosophy*. New York et al.: Longmans, Green, and Co.

James, W. (1931). Principles of

Psychology (Vol. 1). New York: Henry Holt and Company.

Kloos, G. (1938). Störungen des Zeiterlebens in der endogenen Depression, *Der Nervenarzt*, 11, 225-244.

Koffka, K. (1935). *Principles of Gestalt Psychology*. New York: Harcourt, Brace and Co.

Koffka, K. (1928). *The Growth of the Mind*, London: Kegan Paul, Trench, Trübner & Co.

Lewis, A. (1932). The Experience of Time in Mental Disorder. *Proceedings of the Royal Society of Medicine*, 25(5), 611-620.

Mayer-Gross, W. (1935). On Depersonalization. *British Journal of Medical Psychology*, 15, Part 2, 103-126.

Mayor's Committee on Marihuana. (1944). *The Marihuana Problem in the City of New York; Sociological, Medical and Pharmacological Studies*. Lancaster, Pa.: The Jaques Cattell Press,74-75.

Minkowski, E. (1933). Le problème du temps en psychopathologie, *Recherches philosophiques*, 2, 231-256.

Minkowski, E. (1933). *Le temps vecu*. Paris: J. L. L. d'Astrey.

Mueller, G. (1946). Experiential and Existential Time. *Philosophy and Phenomenological Research*, 6(3), 424-435.

Schilder, P. (1942). *Mind: perception and thought in their constructive aspects*. New York: Columbia University Press.

Straus, E. (1928). Das Zeiterlebnis in der endogenen Depression und in der psychopathischen Verstimmung. *Monatsschrift für Psychiatrie und Neurologie*, 68, 126–140.

Straus, E. (1930). Die Formen des Räumlichen. *Der Nervenarzt*, 3(11), 633–656.
